# Determinants of cerebral microbleed presence and burden in CADASIL

**DOI:** 10.1016/j.cccb.2026.100547

**Published:** 2026-05-26

**Authors:** Jessica Lebenberg, Louis Lambert, Laura Tintoré-Carbonell, Mohamed Saichi, Antoine Guillonet, Hugues Chabriat

**Affiliations:** a Inserm 1127, Institut du Cerveau, ICM, F-75013, Paris, France; b Centre Neurovasculaire Translationnel – Centre de référence CERVCO, DMU Neurosciences, FHU NeuroVasc 2030, Hôpital Lariboisiere, APHP, Paris, France; c Service de Neuroradiologie, Hôpital Lariboisiere, APHP, Paris, France; d Université Paris-Cité, France

**Keywords:** Cerebral microbleeds, MRI, Hurdle model, White matter hyperintensities, Lacunes, CADASIL, Small vessel disease

## Abstract

**Background & aims:**

Cerebral microbleeds (CMBs) are observed in one-third of CADASIL patients and increase with age. Factors associated with CMBs vary across studies, likely due to inadequate handling of their zero-inflated, long-tailed distribution and lack of accounting for measurement variability. We aimed to assess these factors while addressing these limitations.

**Methods:**

CMBs were assessed in a large CADASIL cohort, with demographic, vascular, genetic, imaging, and clinical data. White matter hyperintensity volume (WMH), lacune count, and brain parenchymal fraction (BPF) were measured. A Hurdle model was used to distinguish factors associated with CMB presence from those related to lesion counts, while accounting for measurement variability. Three models were built with: (1) demographic/biological factors; (2) MRI markers; and (3) clinical variables.

**Results:**

Beyond the consistent effect of the MRI sequence, in Model 1, CMB presence was associated with age, male sex and hypertension, while counts were associated only with age. In Model 2, CMB presence was associated with age, hypertension, and lacune count; counts were higher with WMH and antiplatelet use, and lower with EGFr 1–6 mutations and BPF. In Model 3, CMB presence was associated with age, hypertension, stroke history and gait disturbances; counts were associated with education, lacune count, MMSE ≤24, and hypercholesterolemia measured at the study visit.

**Conclusions:**

Hurdle modeling distinguishes determinants of CMB occurrence from accumulation. Hypertension appears to lower the threshold for lesion emergence along aging. CMB burden is associated with the burden of cerebrovascular lesions, brain atrophy and clinical severity, supporting worsening of the microangiopathic process, with specific impact related to mutation type.

## Highlights

• Hurdle modeling separates factors of CMB occurrence from MRI marker accumulation.
• Biological drivers of CMB emergence differ from those of their accumulation.
• CMBs as markers of cumulative vascular injury rather than isolated hemorrhage.

## Introduction

Cerebral autosomal dominant arteriopathy with subcortical infarcts and leukoencephalopathy (CADASIL) is a severe monogenic cerebral small vessel disease caused by cysteine-altering mutations in the NOTCH3 gene [1]. The disease leads to recurrent stroke, migraines with aura, and progressive neurological disability, including gait impairment, mood disturbances, and cognitive decline [2,3]. Brain Magnetic Resonance Imaging (MRI) shows multiple tissue lesions throughout the disease course, including white matter hyperintensities (WMH), lacunes, and cerebral microbleeds (CMBs). According to the STRIVE consensus, CMBs correspond to small hemosiderin deposits detected on T2*-weighted or susceptibility-weighted imaging and reflect underlying small-vessel fragility [4,5].

In CADASIL, CMBs are observed in approximately one-third of symptomatic patients, increase with age, and have been associated with hypertension, mutation location, and intracerebral hemorrhage risk [[6], [7], [8], [9]]. Although they are not consistently correlated at cross-sectional level with clinical severity, CMBs are considered markers of advanced vessel wall pathology and may provide insight into disease progression and hemorrhagic vulnerability [10].

However, methodological limitations continue to hamper the identification of factors associated with CMB occurrence and burden, potentially undermining the robustness of previous findings. CMBs are visually defined lesions whose detection depends on MRI acquisition parameters and reader expertise [11,12]. Despite the development of standardized rating tools such as the Microbleed Anatomical Rating Scale (MARS) scale, substantial inter- and intra-rater variability persists, even after additional grading as the Brain Observer MicroBleed Scale (BOMBS) system [12,13]. Yet, this inherent variability has not been formally integrated into the analytical frameworks of prior studies. As a result, prevalence estimates vary widely across different cohorts, and reported associations with clinical or imaging markers appear mainly inconsistent [6,8,[14], [15], [16]]. Second, CMB counts exhibit a highly zero-inflated and overdispersed distribution. Many patients have no detectable lesions, whereas a minority present with multiple microbleeds. Analytical strategies frequently used in prior studies—dichotomization (presence vs absence), simple modeling of raw or log-transformed counts, or categorization into ordinal classes—may therefore inadequately reflect the underlying data structure. Dichotomization is known to reduce statistical power and attenuate effect estimates [17], while categorization introduces information loss and dependence on arbitrary cut-points [18]. Moreover, modeling total counts without separating lesion occurrence from lesion accumulation implicitly assumes that the same determinants govern both processes. Yet in CADASIL, progressive NOTCH3 extracellular domain aggregation and vessel wall dysfunction [19] may influence the initiation of the first CMB differently from the subsequent increase in lesion number. Failure to distinguish these components may also contribute to heterogeneous findings across studies.

Addressing these analytical limitations is essential to understand the determinants of CMBs in CADASIL. In the present study, we first quantified the reliability of CMB identification and counting in a large French CADASIL cohort. We then applied a Hurdle modeling framework that separately evaluates (1) the factors associated with exhibiting at least one CMB and (2) those related to the number of lesions among affected individuals. This two-part approach allows us to determine whether the occurrence of CMBs and their subsequent increase are driven by the same or by distinct biological, clinical, genetic, and imaging factors. By explicitly accounting for measurement variability and the zero-inflated distribution of CMB counts, we aimed to provide more robust and informative estimates of the predictors of CMB presence and burden in CADASIL.

## Materials and methods

### Patients

Patients with CADASIL have been prospectively recruited since 2003 at the French National Referral Centre for Rare Cerebral and Retinal Vascular Diseases (CERVCO), Paris, France (https://www.cervco.fr). The diagnosis was confirmed in all cases by genetic testing demonstrating a pathogenic NOTCH3 variant resulting in an altered number of cysteine residues.

At each visit (every 2 years when possible), standardized demographic, vascular, genetic, clinical, and biological data were collected at the individual level. Recorded variables included age, sex, weight, years of education, and NOTCH3 mutation location (EGFr domains 1–6 vs 7–34). Vascular risk factors were defined as follows: history of hypertension (prior diagnosis or hypotensor medication), hypertension at the time of the visit (systolic blood pressure > 140 mmHg and/or diastolic blood pressure > 90 mmHg at examination), history of diabetes mellitus (prior diagnosis or antidiabetic medication), history of hypercholesterolemia (prior diagnosis or statins medication), hypercholesterolemia at each visit (total cholesterol > 240mg/dL and/or low cholesterol > 160 mg/dL), smoking status (current + former) with cumulative exposure expressed in pack-years, and current or former alcohol use. Additional medical history included prior brain injury. Ongoing treatments were also documented, including antiplatelet agents, as well as other medications (e.g., antidepressants, analgesics). Laboratory parameters comprised hemoglobin, platelet and leukocyte counts, triglycerides, glycemia, fibrinogen, C-reactive protein (CRP), and glycated hemoglobin HbA1c levels.

Clinical outcomes were systematically assessed, including history of stroke, migraine with aura, and gait disturbances. Functional and cognitive status were evaluated by trained neurologists using standardized scales, including the modified Rankin Scale (mRS) and the Mini-Mental State Examination (MMSE).

Informed consent was obtained from each subject or a close relative if the subject was too severely disabled to give written consent. This study was approved by an independent ethic committee (updated agreement CEEI-IRB-17/388) and conducted in accordance with the Declaration of Helsinki.

### MRI acquisition

All participants underwent standardized brain MRI including 3D T1-weighted, FLAIR, and susceptibility-sensitive sequences (T2*-weighted gradient-echo or susceptibility-weighted imaging [SWI]). Detailed acquisition parameters are provided in Table 1.

**Table 1:** **MRI settings**. MRI setting used for the acquisitions. The major point to note is the change of scanner in 2014: between 2003 and 2014, patients underwent T2star MRI within a 1.5T scanner, after 2014, they underwent SWI MRI within a 3T scanner.

	Years of acquisition		2003 – 2014		2014 - 2024	
	Scanner		GE Signa		Siemens	
	Magnetic Field (T)		1.5		3	
	T1w	FLAIR	T2star	T1w	FLAIR	SWI
Pixel spacing (mm^2^)	1.0 × 1.0	0.5 × 0.5	0.9 × 0.9	0.4 × 0.4 1.0 × 1.0	0.5 × 0.5 1 × 1	0.4 × 0.4
Slice Thickness (mm)	1.6	5.5	5.5	0.4 1	0.9 - 1.1	1.5 2
Spacing between slices (mm)	0.8	5.5	5.5	-	-	-
Acquisition Matrix (voxels)	256×256	256×160	256×192	224×224 288×288 320×320	224×164 256×192 256×256	256×190 256×200 288×224
Repetition Time (ms)	9000	8400	500 - 600	1800–2200	5000–6000	22 - 27
Echo Time (ms)	1.9	140 - 160	15	2.3 - 3.2	375 - 395	15 20
Inversion Time (ms)	0	210	0	900 - 910	1600 - 1900 2200	-
Flip angle (°)	20	90	20	8	120	15
Band width/pixel (Hz/pixel)	122	61	122	200 - 330	700 - 815	120

Importantly, MRI hardware and susceptibility sequences evolved during the study period. Between 2003 and 2014, examinations were performed on a 1.5T GE scanner including T2*-weighted gradient-echo imaging for microbleed detection. From 2014 onward, scans were acquired on a 3T Siemens system including SWI. Because both magnetic field strength and susceptibility sequence influence microbleed detectability, the imaging sequence was explicitly accounted for in all statistical analyses.

### Cerebral microbleeds: identification and reliability assessment

**Fig. 1:**
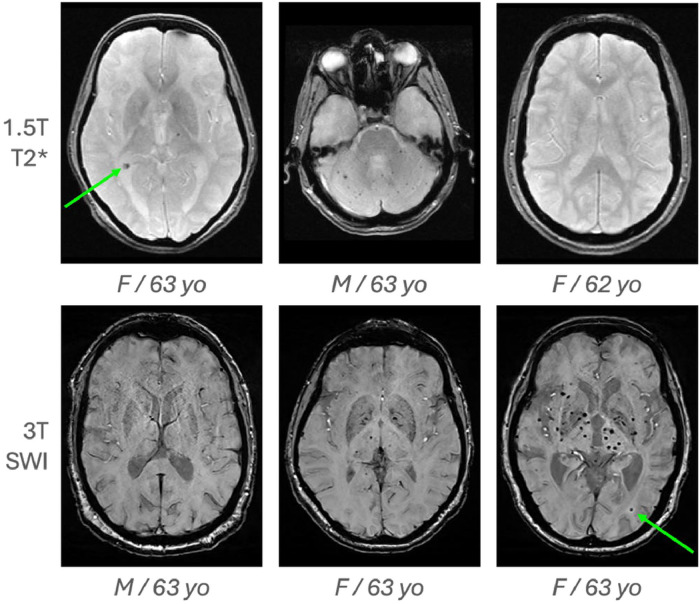
**CMB identification**. T2* scans (first row) and SWI scans (second row) from different of individuals of male (M) or female (F) sex, at different age. Arrows indicate microbleeds identified visually.

Cerebral microbleeds (CMBs) were the primary imaging outcome. CMBs were visually identified on each susceptibility-sensitive sequence (T2*-weighted gradient echo or SWI) by a single highly trained scientific rater, blinded to all clinical and biological data, according to the STRIVE consensus criteria [4,5] (Fig. 1). Care was taken to differentiate true CMBs from vascular flow voids, calcifications, and susceptibility artefacts. The total number of CMBs was automatically recorded for each participant at each visit.

Given the known variability in visual CMB detection and the change in MRI sequence during the study period, a formal reliability analysis was conducted. Sixty MRI scans were randomly selected from the full database: 30 acquired with T2*-weighted imaging and 30 with SWI. In the T2* subgroup, the mean age was 60.2 ± 9.0 years and 63% were women; in the SWI subgroup, the mean age was 58.9 ± 7.8 years and 50% were women.

CMBs were independently identified twice by the same experienced reader (Review 1 and Review 2), blinded to prior ratings and weeks apart. The total number of CMBs was automatically computed after manual marking. Counts were analyzed both as continuous values and as binary variables (absence vs presence).

Binary agreement was evaluated using weighted Cohen’s kappa [20]. Continuous agreement was assessed using a two-way mixed-effects intraclass correlation coefficient (ICC3) and paired Wilcoxon signed-rank testing to detect systematic differences between readings [21,22]. To quantify the impact of lesion burden on counting variability, the absolute difference between readings was modeled as a function of the mean CMB count and MRI sequence (T2* vs SWI).

A normalized error rate (absolute difference divided by the mean count) was also calculated and plotted against the mean lesion burden. A clinically acceptable error threshold was predefined at 20%, allowing determination of the maximal CMB count for which visual quantification remained reliable (Supp Materials, Supp Figure 1). This reliability-informed threshold was subsequently incorporated into the analytical framework.

### Associated imaging markers

White matter hyperintensities (WMH) were segmented using validated automated pipelines: either the LST-LPA algorithm within the SPM/Matlab framework applied to native FLAIR images, or the BIANCA algorithm from the FSL suite applied to preprocessed FLAIR and T1-weighted images [[23], [24], [25], [26]] (Supp Materials, Appendix A). Probabilistic lesion maps were thresholded according to established procedures, and all WMH masks were systematically reviewed and manually corrected by an experienced rater.

Lacunes were segmented at baseline as previously described [27]. Briefly, regions of interest were defined on T1-weighted images as clusters of voxels exhibiting CSF-like signal intensity within the deep white matter and meeting STRIVE-based diameter criteria [4,5]. All lacunar masks were visually inspected and corrected by the same expert.

Global brain atrophy was quantified from T1-weighted images. Intracranial cavity (ICC) and cerebrospinal fluid (CSF) masks were segmented using the ANTs pipeline ([28]; [29]). Brain tissue volume was calculated by subtracting CSF volume from ICC volume. The brain parenchymal fraction (BPF) was computed as: BPF = brain tissue volume / intracranial volume × 100, providing a normalized measure of whole-brain tissue loss.

### Statistical analysis

The analysis of descriptive covariates associated with CMB emergence and increase was performed on all inclusion visits available in the cohort (Table 2). Among these scans, one T1-weighted MRI and two FLAIR sequences were not suitable for analysis, and one patient did not undergo T2*-weighted or SWI imaging for CMB assessment. Biological markers with >10% missing data (some reaching up to 25%) were excluded from the set of potential predictors. All continuous variables were min-max normalized to scale between 0 and 1 for the subsequent analysis.

**Table 2:** **Main characteristics of the cohort at baseline**. Description of the cohort at the baseline visit: for the whole group (left column), for the group of patients without any microbleed (CMB) (middle column), and with at least one CMB (right column). Continuous variables are presented with the mean value ± standard deviation (n values available, n missing values). Binary variables are presented by the n positive values / n available values (%, n missing values). Univariate comparisons have been performed between the group with and without microbleeds using a Mann-Whitney U test (two-sided) for continuous variables, and a Pearson chi-square test on contingency table for binary variables. P-value thresholds are : * if p < 0.05, ** if p < 0.01, *** if p < 0.001. Abb :[N : number of patients ; SWI : SWI MRI sequence used to identified microbleeds]; Demographic :[ N Years Education : number of years of education ; EGFr 1–6 : genetic mutation located in EGFr 1–6];Risk factors : [ Smocking : smoking status (current or former) ; N PackCigarettes Year : number of pack of cigarettes-years ; Diabetes : history of diabetes mellitus (prior diagnosis or antidiabetic medication) ; HTA History : history of hypertensive status of the patient (and/or hypertensor medication); HTA Visit : hypertension at the time of the visit (systolic blood pressure > 140 mmHg and/or diastolic blood pressure > 90 mmHg at examination), Hypercholesterolemia History : history of hypercholesterolemia (prior diagnosis or statins medication), Hypercholesterolemia Visit : hypercholesterolemia at each visit (total cholesterol > 240mg/dL and/or low cholesterol > 160 mg/dL) ; Heart failure : history of heart failure of the patient ; Brain Injury : history of brain injury of the patient]; Treatments : use of medications ; Biology : levels in biology prelevments ; MRI Markers : [N Lacunes : number of lacunes; nWMH : white matter hyperintensity volume normalized by the total intra-cranial cavity; BPF : brain parenchymal fraction ; Clinical : [Stroke / Migraine with aura / Gait Disturbance :history of events ; mrS ≥ 3 : modified Rankin Scale binarized – threshold at 3 – ; MMSE ≤ 24 : Mini-Mental State Examination binarized – threshold at 24 –].

Variable	Overall	CMB absent	CMB present	p-value
N	525	325	199	
SWI	233/524 (44.5%, NA=1)	122/325 (37.5%, NA=0)	111/199 (55.8%, NA=0)	<0.001***
*Demographic*				
Male Sex	245/525 (46.7%, NA=0)	142/325 (43.7%, NA=0)	103/199 (51.8%, NA=0)	0.088
Weight	73.6 ± 15.2 (n = 512, NA=13)	71.9 ± 14.8 (n = 319, NA=6)	76.5 ± 15.5 (n = 193, NA=6)	0.001**
N Years Education	11.7 ± 4.1 (n = 513, NA=12)	12.1 ± 4.0 (n = 323, NA=2)	11.0 ± 4.1 (n = 190, NA=9)	0.010*
Age	52.4 ± 12.1 (n = 525, NA=0)	49.0 ± 11.9 (n = 325, NA=0)	58.0 ± 10.1 (n = 199, NA=0)	<0.001***
EGFr 1–6	312/525 (59.4%, NA=0)	220/325 (67.7%, NA=0)	91/199 (45.7%, NA=0)	<0.001***
*Risk Factors*				
Smoking	251/525 (47.8%, NA=0)	164/325 (50.5%, NA=0)	87/199 (43.7%, NA=0)	0.159
N PackCigarettes Year	8.8 ± 14.4 (n = 510, NA=15)	9.6 ± 15.4 (n = 313, NA=12)	7.7 ± 12.6 (n = 196, NA=3)	0.195
Diabetes	28/525 (5.3%, NA=0)	12/325 (3.7%, NA=0)	16/199 (8.0%, NA=0)	0.051
Alcohol	390/525 (74.3%, NA=0)	233/325 (71.7%, NA=0)	156/199 (78.4%, NA=0)	0.110
HTA History	148/525 (28.2%, NA=0)	61/325 (18.8%, NA=0)	87/199 (43.7%, NA=0)	<0.001***
HTA Visit	129/525 (24.6%, NA=0)	58/325 (17.8%, NA=0)	70/199 (35.2%, NA=0)	<0.001***
Hypercholesterolemia History	244/525 (46.5%, NA=0)	138/325 (42.5%, NA=0)	105/199 (52.8%, NA=0)	0.027*
Hypercholesterolemia Visit	105/525 (20.0%, NA=0)	72/325 (22.2%, NA=0)	33/199 (16.6%, NA=0)	0.152
Heart failure	5/525 (1.0%, NA=0)	4/325 (1.2%, NA=0)	1/199 (0.5%, NA=0)	0.712
Brain Injury	31/525 (5.9%, NA=0)	14/325 (4.3%, NA=0)	16/199 (8.0%, NA=0)	0.112
*Treatments*				
Anti Platelets	65/525 (12.4%, NA=0)	36/325 (11.1%, NA=0)	29/199 (14.6%, NA=0)	0.298
Antidepressant	81/525 (15.4%, NA=0)	44/325 (13.5%, NA=0)	37/199 (18.6%, NA=0)	0.153
Analgesics	33/525 (6.3%, NA=0)	20/325 (6.2%, NA=0)	13/199 (6.5%, NA=0)	1.000
*Biology*				
Hemoglobin	14.1 ± 1.4 (n = 519, NA=6)	14.0 ± 1.3 (n = 321, NA=4)	14.3 ± 1.5 (n = 197, NA=2)	0.002**
Leukocyte	6.4 ± 2.6 (n = 519, NA=6)	6.4 ± 3.1 (n = 321, NA=4)	6.4 ± 1.7 (n = 197, NA=2)	0.158
Platelet	252.7 ± 61.1 (n = 519, NA=6)	254.3 ± 60.5 (n = 321, NA=4)	250.5 ± 61.9 (n = 197, NA=2)	0.431
Triglycerides	1.2 ± 0.6 (n = 514, NA=11)	1.1 ± 0.7 (n = 321, NA=4)	1.2 ± 0.6 (n = 192, NA=7)	0.019*
C-reactive protein	5.2 ± 6.4 (n = 501, NA=24)	5.3 ± 7.5 (n = 312, NA=13)	5.0 ± 4.2 (n = 188, NA=11)	0.365
Glycated hemoglobin HbA1c	5.5 ± 0.6 (n = 509, NA=16)	5.5 ± 0.4 (n = 317, NA=8)	5.7 ± 0.7 (n = 191, NA=8)	<0.001***
Fasting Glycemia	5.4 ± 1.1 (n = 513, NA=12)	5.3 ± 0.9 (n = 318, NA=7)	5.5 ± 1.2 (n = 194, NA=5)	0.011*
Fibrinogen	3.3 ± 0.7 (n = 494, NA=31)	3.2 ± 0.7 (n = 304, NA=21)	3.4 ± 0.7 (n = 189, NA=10)	0.019*
*MRI Markers*				
N Lacunes	9.1 ± 10.6 (n = 524, NA=1)	6.1 ± 9.0 (n = 325, NA=0)	14.1 ± 11.3 (n = 198, NA=1)	<0.001***
nWMH	5.3 ± 4.0 (n = 522, NA=3)	4.8 ± 3.9 (n = 324, NA=1)	6.1 ± 4.1 (n = 198, NA=1)	<0.001***
BPF	81.9 ± 4.3 (n = 524, NA=1)	83.0 ± 4.1 (n = 325, NA=0)	80.1 ± 4.1 (n = 198, NA=1)	<0.001***
*Clinical*				
Stroke	260/525 (49.5%, NA=0)	124/325 (38.2%, NA=0)	135/199 (67.8%, NA=0)	<0.001***
Migraine With Aura	207/525 (39.4%, NA=0)	153/325 (47.1%, NA=0)	54/199 (27.1%, NA=0)	<0.001***
Gait Disturbance	141/525 (26.9%, NA=0)	50/325 (15.4%, NA=0)	90/199 (45.2%, NA=0)	<0.001***
mRS ≥ 3	77/522 (14.8%, NA=3)	26/323 (8.0%, NA=2)	50/198 (25.3%, NA=1)	<0.001***
MMSE ≤ 24	96/489 (19.6%, NA=36)	44/311 (14.1%, NA=14)	52/178 (29.2%, NA=21)	<0.001***

Three hierarchical models were constructed. Model 1 included only demographic and biological variables, as well as the type of MRI sequence used for CMB identification (T2* or SWI). Model 2 additionally incorporated MRI biomarkers (BPF, WMH normalized by the intracranial cavity [nWMH], and number of lacunes). Model 3 further included clinical outcomes (history of migraine with aura, stroke and gait disturbance) and binarized clinical scores (mRS ≥ 3, yes/no; MMSE ≤ 24, yes/no). For each model, analyses were performed on complete cases only; visits with missing values were excluded.

For each model, variable selection was carried out using a Lasso-based approach, with the optimal regularization parameter (λ) determined by five-fold cross-validation for both continuous (Lasso regression) and binary (logistic L1-penalized) outcomes [[30], [31], [32]]. To ensure stability of the selection process, only the top 15 variables with non-zero Lasso coefficients were retained.

**Fig. 2:**
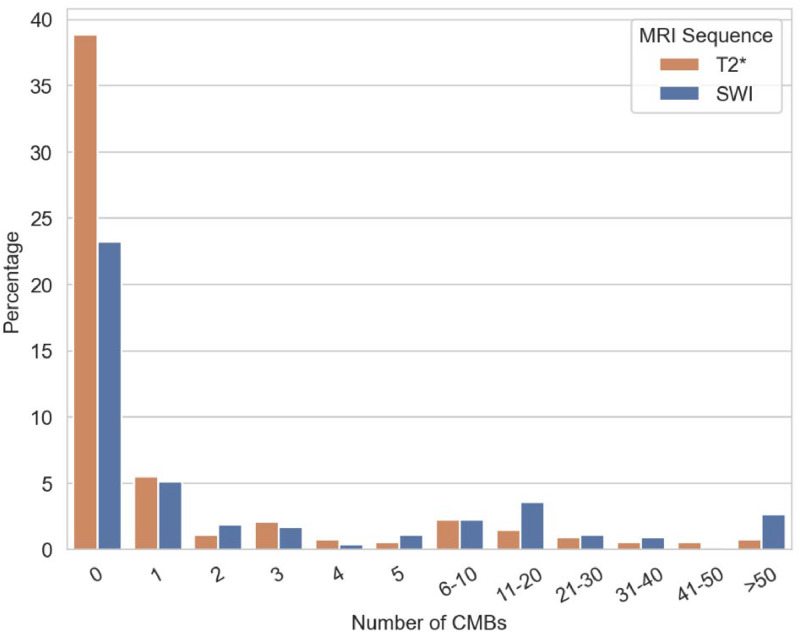
**CMB distribution in the whole cohort at the baseline visit**. Zero-inflated distribution of CMB counts according to MRI sequence (T2*-weighted acquisitions in orange, SWI in blue). Regardless of the sequence, the distribution is characterized by an excess of zeros and a long tail.

Given the zero-inflated and long-tailed distribution of CMB counts (Fig. 2), a two-step Hurdle framework was applied. First, a logistic regression modeled CMB absence versus presence. Second, CMB counts among patients with at least one lesion were analyzed. The dispersion index (variance/mean) was computed, and depending on its value, either a Poisson or quasi-Poisson distribution was selected [[33], [34], [35], [36]].

The Hurdle model was refined by retaining variables with p-values < 0.2 after the initial estimation. The final model was validated by assessing collinearity using the variance inflation factor (VIF) and removing major sources of collinearity when necessary. Forest plots were generated to present the final odds ratios (ORs) and incidence rate ratios (IRRs).

To assess robustness while accounting for potential CMB measurement errors, a sensitivity analysis was performed using 100 simulations. In each simulation, CMB counts were modulated within the predefined acceptable clinical error margin (20%). The Hurdle framework with the final set of variables was re-estimated, and mean ORs/IRRs with corresponding mean confidence interval limits were calculated.

All analysis were performed under Python v3.12.11 and using pandas (v2.3.3), numpy (v2.3.3), scipy (v1.16.3), statsmodels (V0.14.5), and scikit-learn (v.1.7.2) packages [31,37]. Graphs were plotted with seaborn (v0.13.2), and matplotlib (v3.10.6) [38].

## Results

### Reliability analysis of CMB counting in CADASIL

#### Binary agreement (Absence/Presence)

For binary classification (absence vs presence of CMBs), agreement between readings was excellent. The weighted kappa coefficient reached 1.00 for the T2*-weighted sub-dataset and 0.90 for the SWI sub-dataset. Only one scan among the 30 SWI examinations was discordant.

#### Continuous agreement (CMB counts)

For continuous CMB counts, the ICC3 was 0.95 for T2*-weighted scans and 0.87 for SWI scans, indicating high reproducibility. Paired comparisons showed no significant difference between the two readings for the T2* sub-dataset, whereas a statistically significant difference was observed for SWI scans. Nevertheless, 60% of SWI paired counts were strictly identical (difference = 0) (Supp Materials, Supp Figure 2).

**Fig. 3:**
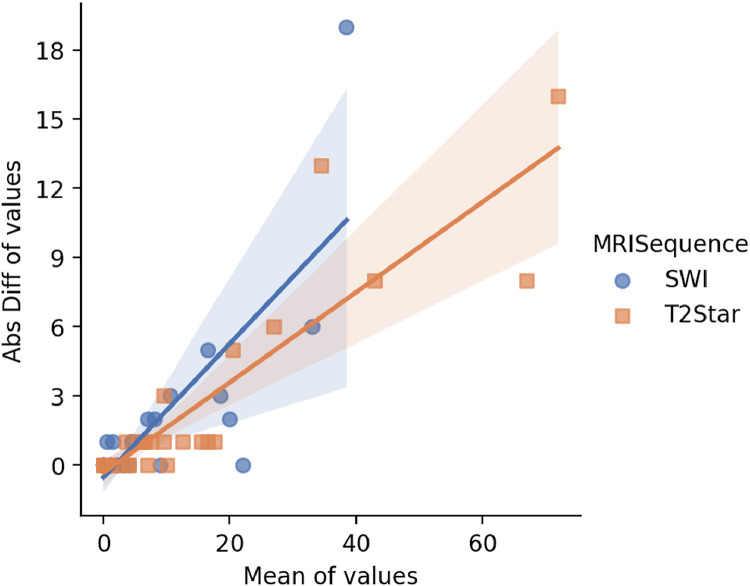
**Absolute differences in CMB counts between two readings, relative to the mean count, according to MRI sequence**. Absolute differences in CMB counts between the two readings, relative to the mean count, are shown for the SWI (blue) and T2*-weighted (orange) sub-datasets. In both sub-datasets, differences increased with higher mean counts, regardless of the MRI sequence used for rating.

**Fig. 4:**
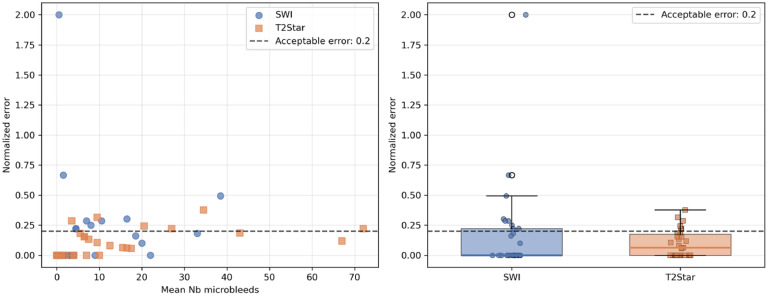
**Normalized error regards to the mean value of the counting and the MRI sequence**. Scatter plot (left) and boxplot (right) of the normalized errors (absolute difference / mean counting) for the SWI (blue) and the T2* (orange) dataset. The boxplot representations showed that >75% and close to 75% of the errors were less than the acceptable clinical error fixed at 20% (dotted line).

Multivariate modeling of the absolute counting difference as a function of the mean CMB count and MRI sequence demonstrated that counting variability was significantly associated with lesion burden, regardless of the imaging sequence used (Table 3; Fig. 3). In contrast, the normalized error (absolute difference divided by mean count) was independent of mean lesion burden, and approximately 75% of errors were below the predefined acceptable clinical threshold of 20% (Fig. 4).

**Table 3:** **Multivariate modeling of the absolute counting difference as a function of the mean CMB count and MRI sequence**. Modeling of the absolute difference in microbleeds (CMB) counting as a function of the mean counting value and the MRI sequence (Mean Nb CMBs couting). The absolute difference in CMBs counting was significatively dependent of the mean couting (palue < 0.05).

Dep. Variable	Abs. Diff CMBs counting		R-squared		0.709
Model	OLS		Adj. R-squared		0.699
Method	Least Squares		F-statistic		12.13
Nb observations	60		Prob (F-statistic)		4.10e-05
Df residuals	57		Log-Likelihood		−128.28
Df model	2		AIC		262.6
Covariance type	HC3		BIC		[0.025–268.8
	coef	std err	t	P>|t|	0.975]
Intercept	−0.0140	0.366	−0.038	0.970	−0.746–0.718
MRI sequence [T.T2Star]	−0.6035	0.682	−0.885	0.380	−1.969–0.762
Mean Nb CMBs couting	0.2169	0.046	4.677	**0.000**	0.124–0.310

### Factors associated with CMB emergence and burden in CADASIL

Given the marked overdispersion in CMB counts (variance/mean > 80), a quasi-Poisson regression was used to model CMB burden in all models. All results are displayed in Supp Materials, Supp Table 1 and Supp Figure 3. For continuous covariates, coefficients represent the effect associated with an increase from the minimum to the maximum observed value (variables were scaled by min–max normalization).

#### Model 1: demographic and biological covariates

In Model 1, 424 patients were included in the logistic component (modeling CMB presence), and 156 patients with ≥1 CMB were included in the quasi-Poisson component (modeling CMB burden).

For the logistic component, variables retained for analysis were age, sex, mutation location, history of hypertension, and MRI sequence (T2* vs SWI). No collinearity was detected (all VIF < 5). Higher age, male sex, a history of hypertension, and the use of SWI were significantly associated with the presence of CMBs.

For the quasi-Poisson component, variables retained for analysis were age, MRI sequence, hypercholesterolemia (history and measured at the study visit), and mutation location. No collinearity was observed (all VIF < 5). Higher age and the use of SWI were significantly associated with higher CMB counts.

#### Model 2: demographic, biological, and MRI biomarkers

In Model 2, 423 patients were included in the logistic regression and 155 in the quasi- Poisson component.

For CMB presence, variables retained for analysis were lacune count, age, history of brain injury, hypercholesterolemia and hypertension, MRI sequence, mutation location, and cumulative smoking exposure (pack-years). No collinearity was detected (all VIF < 5). Higher lacune count, higher age, a history of hypertension, and the use of SWI were significantly associated with the presence of CMBs.

For CMB burden, variables retained for analysis were normalized age, WMH volume, MRI sequence, number of lacunes, antiplatelet therapy, hypercholesterolemia measured at the study visit, mutation location, and brain parenchymal fraction (BPF). The variable Age got a VIF at 6.3 and was collinear with the WMH volume (0.49), the number of lacunes (0.31) and the BPF (−0.61). We thus removed from the analysis this variable and run again the model. No more collinearity was detected afterwards. Higher normalized WMH volume, use of SWI, and antiplatelet therapy were significantly associated with higher CMB counts. In contrast, mutation located in EGFr 1–6 and higher BPF were associated with lower CMB counts.

#### Model 3: including binarized cognitive scores

In Model 3, 400 patients were included in the logistic component and 140 in the quasi-Poisson component.

For CMB presence, variables retained for analysis were age, history of hypertension and stroke, MRI sequence, mutation location, dichotomized mRS, and gait disturbance. No collinearity was detected (all VIF < 5). Older age, a history of hypertension and stroke, the presence of gait disturbance, and the use of SWI were significantly associated with the presence of CMBs. Mutation located in EGFr 1–6 was associated with a lower risk of CMB presence.

For CMB burden, variables retained for analysis were years of education, lacune count, dichotomized MMSE (≤ 24), hypercholesterolemia measured at the study visit, mutation location, and migraine with aura. No collinearity was observed (all VIF < 5). Higher education, greater lacune count, MMSE ≤ 24, and hypercholesterolemia measured at the study visit were significantly associated with higher CMB counts. In contrast, migraine with aura and mutation located in EGFr 1–6 were associated with lower CMB burden.

**Fig. 5:**
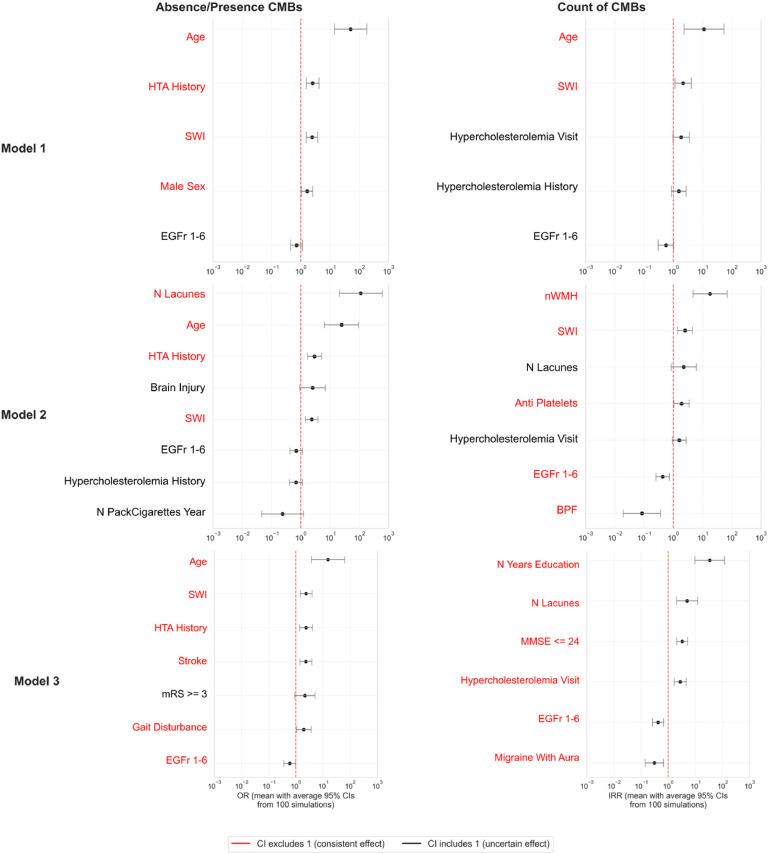
**Variables associated with CMB emergence and burden in CADASIL across the 3 different models**. Forest plots of the odd ratios OR (left) and incidence rate ratios IRR (right) scores obtained for the logistic (absence/presence) and quasi-poisson (count) regressions in the frame of the validation of the different models. For a better visualization, results were plotted with a log scale. Variables in red were significantly associated with the presence or increase in number of cerebral microbleeds. Abb : SWI : SWI MRI sequence used to identified microbleeds; N Years Education : number of years of education; EGFr 1–6 : genetic mutation located in EGFr 1–6; N PackCigarettes Year : number of pack of cigarettes-years; HTA History : history of hypertensive status of the patient (and/or hypertensor medication); Hypercholesterolemia History : history of hypercholesterolemia (prior diagnosis or statins medication); Hypercholesterolemia Visit : hypercholesterolemia at each visit (total cholesterol > 240mg/dL and/or low cholesterol > 160 mg/dL); Brain Injury : history of brain injury of the patient; Anti Platelets : anti-platelets medication; N Lacunes : number of lacunes; nWMH : white matter hyperintensity volume normalized by the total intra-cranial cavity; BPF : brain parenchymal fraction; Stroke / Migraine with aura / Gait Disturbance : history of events; mrS ≥ 3 : modified Rankin Scale binarized – threshold at 3 –; MMSE ≤ 24 : Mini-Mental State Examination binarized – threshold at 24 –.

All these models were validated across 100 simulated datasets incorporating a ± 20% measurement error in CMB counts (Table 4 and Fig. 5).

**Table 4:** **Validation of associated variables of CMB Emergence and Increase in CADASIL in different models**. Mean odd ratio OR (resp. mean incidence rate ratios IRR obtained for the logistic – absence/presence – (resp. quasi-poisson – count –) regressions in the frame of validation of the different models. The 2.5th and 97.5th percentiles represent the empirical 95 % intervals. Significant associations (intervals not including 1) are indicated in bold (p < 0.05 by empirical criterion). Abb : SWI : SWI MRI sequence used to identified microbleeds; N Years Education : number of years of education ; EGFr 1–6 : genetic mutation located in EGFr 1–6; N PackCigarettes Year : number of pack of cigarettes-years ; HTA History : history of hypertensive status of the patient (and/or hypertensor medication); Hypercholesterolemia History : history of hypercholesterolemia (prior diagnosis or statins medication) ; Hypercholesterolemia Visit : hypercholesterolemia at each visit (total cholesterol > 240mg/dL and/or low cholesterol > 160 mg/dL) ; Brain Injury : history of brain injury of the patient; Anti Platelets : anti-platelets medication ; N Lacunes : number of lacunes; nWMH : white matter hyperintensity volume normalized by the total intra-cranial cavity; BPF : brain parenchymal fraction ; Stroke / Migraine with aura / Gait Disturbance :history of events ; mrS ≥ 3 : modified Rankin Scale binarized – threshold at 3 – ; MMSE ≤ 24 : Mini-Mental State Examination binarized – threshold at 24 –.

	OR/IRR	2.5%	97.5%
*Model 1 (demographic, biological co-variates)*			
*Absence / Presence*			
Age	**50.23**	**14.21**	**177.75**
HTA History	**2.52**	**1.53**	**4.15**
SWI	**2.41**	**1.52**	**3.81**
Male Sex	1.63	1.04	2.55
EGFr 1–6	0.71	0.45	1.13
*COUNT*			
Age	**11.13**	**2.30**	**53.92**
SWI	**2.14**	**1.13**	**4.07**
Hypercholesterolemia Visit	1.84	0.97	3.50
Hypercholestereolemia History	1.52	0.86	2.70
EGFr 1–6	0.56	0.30	1.03
*Model 2 (demographic, biological and MRI co-variates)*			
*Absence / Presence*			
N Lacunes	**111.51**	**20.74**	**600.55**
Age	**24.46**	**6.41**	**93.41**
HTA History	**2.93**	**1.69**	**5.09**
Brain Injury	2.48	0.91	6.74
SWI	**2.35**	**1.44**	**3.82**
EGFr 1–6	0.69	0.42	1.13
Hypercholesterolemia	0.67	0.40	1.13
N PackCigarettes Year	0.24	0.05	1.21
*COUNT*			
nWMH	**17.91**	**4.67**	**68.80**
SWI	**2.48**	**1.40**	**4.41**
N Lacunes	2.25	0.83	6.10
Anti Platelets	**1.88**	**1.04**	**3.43**
Hypercholesterolemia Visit	1.57	0.92	2.69
EGFr 1–6	**0.43**	**0.26**	**0.72**
BPF	**0.08**	**0.02**	**0.36**
Model 3 (demographic, Biological, MRI and clinical/Cognitive co-variates (binarized values)			
Absence / Presence			
Age	**15.30**	**3.74**	**62.74**
SWI	**2.39**	**1.45**	**3.92**
HTA History	**2.36**	**1.38**	**4.06**
Stroke	**2.34**	**1.41**	**3.88**
mRS ≥ 3	2.13	0.90	5.06
Gait Disturbance	**1.95**	**1.05**	**3.66**
EGFr 1–6	**0.60**	**0.36**	**0.99**
COUNT			
N Years Education	**33.51**	**9.50**	**118.37**
N Lacunes	**4.89**	**2.00**	**11.97**
MMSE ≤ 24	**3.23**	**2.04**	**5.13**
Hypercholesterolemia Visit	**2.75**	**1.66**	**4.54**
EGFr 1–6	**0.42**	**0.26**	**0.67**
Migraine with aura	**0.30**	**0.14**	**0.66**

## Discussion

In this study, we identified distinct determinants of CMB emergence and accumulation in CADASIL by separating these processes using a hurdle modeling approach. Beyond the differences in MRI detection sensitivity using SWI and T2*, overall, the results indicate that the presence of CMBs is primarily associated with age, hypertension, and lacune load, whereas CMB burden is mainly related to the global severity of small vessel disease, including WMH, brain atrophy, and clinical severity. Importantly, these differences are mainly revealed when structural MRI markers are considered, highlighting the central role of cumulative brain damage in the occurrence and burden of microbleeds.

When not accounting for other MRI markers (Model1), older age and MRI sequence were consistently associated with both CMB presence and burden, while male sex and a history of hypertension were additionally associated with CMB presence. Obviously, the strong effect of MRI sequence, particularly SWI, reflects differences in detection sensitivity rather than biological mechanisms. In contrast, the consistent association with age supports a time-dependent process, whereby progressive structural alterations of the microvascular wall—likely evolving over time and driven by NOTCH3-ECD accumulation—contribute to both the emergence and accumulation of CMBs, as similarly observed in other cohorts [7,16,39,40]. Notably, despite the major impact of age, CMBs were observed only in a subset of patients, indicating that aging and disease progression alone do not fully account for their occurrence. In this context, the associations with hypertension as already observed [7,8,15,41] and male sex also recently reported among Korean patients [39], restricted to CMB presence, suggest that these factors may modulate the early stages of lesion development, possibly influencing the threshold at which microbleeds become detectable during aging. In contrast, the limited number of factors associated with CMB burden from the outset indicates that, without accounting for structural MRI damage, key determinants of microbleed accumulation remain insufficiently captured.

When structural MRI markers were considered in the analyses (Model2), the pattern of associations changed substantially. For CMB presence, age and MRI sequence remained significant, but hypertension and lacune count clearly emerged as independent determinants, while the effect of male sex disappeared. This finding suggests that shared microvascular alterations may underlie both ischemic and hemorrhagic lesions, and that hypertension accelerates these processes during aging, whereas sex would play a more limited role. For CMB burden, higher WMH volume and lower brain parenchymal fraction were strongly associated with increased CMB counts, indicating that accumulation of microbleeds reflects the global increase of cerebral tissue lesions. In addition, antiplatelet therapy was associated with higher CMB counts as already detected [16], although its interpretation remains difficult given its frequent indication in patients with prior ischemic stroke or small infarcts on MRI. The direct role of antiplatelet therapy in the occurrence of microbleeds cannot be excluded but remains uncertain in this context. Genotype effects further refined this picture, as CMBs were more frequent in patients with mutations outside EGFr domains 1–6, suggesting that susceptibility to microbleeds is not univocally linked to the greater accumulation of NOTCH3-ECD presumed to be associated with this mutation location. This unexpected finding raises the possibility that mutation-specific factors, potentially related to structural or physicochemical properties of the protein accumulating in the vascular wall, may also facilitate the leakage of red blood cells from the vascular lumen into the surrounding tissue. The increased risk of intracerebral hemorrhage reported with specific variants such as R544C is consistent with this interpretation [6,14]. Overall, these results indicate that once lesions have emerged, their accumulation is primarily driven by global vascular disease severity rather than by demographic or vascular risk factors alone.

Finally, when clinical variables were incorporated into the model (Model3), interpretation became more complex due to interdependencies between imaging markers and clinical manifestations. Hypertension, prior stroke, and the occurrence of gait disturbances—typically observed at more advanced disease stages—were associated with CMB presence, whereas cognitive decline and hypercholesterolemia measured at the study visit were associated with higher CMB counts. In contrast, migraine with aura was associated with a lower CMB burden [8], consistent with previous reports; a similar association was observed for mutations in EGFr domains 1–6. These findings further support that CMBs occur preferentially in patients with more advanced disease and severe brain lesions in line with previous reports [7,16,39]. The positive association observed with education level and the hypercholesterolemia measured at the study visit in the final model is difficult to interpret and should be considered with caution, as it may reflect residual confounding or complex, non-independent relationships with clinical and imaging variables.

From a pathophysiological perspective, our findings support a model in which progressive microvascular wall alterations—partly specific and variably expressed—lead first to the emergence of CMBs and subsequently to their accumulation alongside other markers of tissue damage. Microbleeds likely reflect advanced vascular wall impairment, potentially involving degeneration of smooth muscle cells and/or pericytes. Their strong associations with lacunes, WMH, brain atrophy, and clinical severity indicate that they are not isolated hemorrhagic events but rather part of a global microangiopathic process. Hypertension appears to lower the threshold for initial lesion formation rather than to drive subsequent accumulation.

Several methodological aspects strengthen the robustness and interpretability of these findings. First, CMB assessment in this study demonstrated excellent reproducibility across imaging modalities. Inter-rater agreement for the presence versus absence of CMBs was near perfect, with weighted kappa values of 1.00 on T2*-weighted imaging and 0.90 on SWI. Agreement for continuous CMB counts was similarly high, with intraclass correlation coefficients of 0.95 for T2* and 0.87 for SWI, confirming strong consistency across modalities. Although agreement was slightly lower with SWI, this likely reflects its higher sensitivity for detecting small or borderline lesions, which may increase ambiguity in distinguishing true microbleeds from artifacts. Importantly, 60% of SWI paired counts were identical between readings, and overall reliability remained within the excellent range for clinical measurements.

A second major strength lies in the detailed quantitative evaluation of counting variability. As expected, absolute intra-rater differences increased with lesion burden, particularly in patients with numerous small lesions. However, the normalized error—defined as the absolute difference divided by the mean count—remained independent of lesion load. Approximately 75% of discrepancies fell below a predefined 20% acceptable threshold, indicating stable proportional precision even in patients with high CMB counts. This analysis provides important reassurance that CMB quantification remains reliable across the full spectrum of disease severity and offers a practical framework for interpreting changes in CMB counts in both clinical and research settings. Importantly, beyond this detailed characterization, this variability was also incorporated into the statistical analyses when identifying predictors, thereby accounting for a source of measurement uncertainty which has not been considered in previous similar studies.

Finally, the use of a hurdle modeling strategy represents a key methodological contribution. By explicitly separating the determinants of CMB presence from those of lesion burden, this approach allowed identification of distinct and non-overlapping factors associated with lesion emergence and accumulation. Modeling CMBs as a single continuous outcome would likely have obscured these differences and limited mechanistic interpretation. Finally, the integration of demographic, clinical, and imaging variables within a unified analytical framework enabled a comprehensive assessment of CMBs in the context of overall disease severity, strengthening the biological plausibility and coherence of the observed associations.

Several limitations should be acknowledged. The cross-sectional design precludes causal inference and limits conclusions regarding temporal relationships. The study population, derived from a specialized CADASIL cohort, may limit generalizability to other forms of small vessel disease. Information on blood pressure control and treatment exposure was limited, and the association with antiplatelet therapy remains difficult to interpret due to indication bias. CMB topography was not analyzed and will be investigated in the future as it may provide additional mechanistic insights. Cognitive assessment also relied on global measures, which may not capture domain-specific alterations. Finally, direct biomarkers of vascular wall alteration or NOTCH3-ECD accumulation were not available, restricting mechanistic interpretation.

Overall, by distinguishing factors associated with CMB emergence from those related to lesion accumulation and by accounting for measurement variability, this study refines the interpretation of microbleeds in CADASIL. CMBs appear to mark a threshold of vascular wall damage, whereas their accumulation reflects overall disease severity. Their clinical relevance should therefore be considered in conjunction with other MRI markers—particularly lacunes, WMH, and brain atrophy—which more directly drive clinical outcomes.

## Data availability

The data used in this study are available from the corresponding author and co-authors upon reasonable request.

## Declaration of generative AI and AI-assisted technologies in the manuscript preparation process

During the preparation of this work the authors used OpenAI (gpt-5-the-latest) to help to design the study and Anthropic (claude-opus-4–1) to help to code. After using these tools, the authors reviewed and edited the content as needed and take full responsibility for the content of the published article.

## CRediT authorship contribution statement

**Jessica Lebenberg:** Writing – review & editing, Writing – original draft, Visualization, Validation, Methodology, Formal analysis, Data curation, Conceptualization. **Louis Lambert:** Writing – review & editing, Validation, Methodology, Formal analysis, Conceptualization. **Laura Tintoré-Carbonell:** Writing – review & editing, Methodology, Formal analysis, Conceptualization. **Mohamed Saichi:** Writing – review & editing, Visualization. **Antoine Guillonet:** Writing – review & editing, Data curation. **Hugues Chabriat:** Writing – review & editing, Writing – original draft, Validation, Supervision, Project administration, Methodology, Investigation, Formal analysis, Conceptualization.

## Declaration of competing interest

The author(s) declare no commercial or financial relationships that could be construed as a potential conflict of interest.

## Appendix Supplementary materials

Image, application 1
