# Supplementary material for: Determinants of cerebral microbleed presence and burden in CADASIL
Source: Cereb Circ Cogn Behav. 2026 May 26;10:100547. doi: 10.1016/j.cccb.2026.100547 (PMC13241976; doi:10.1016/j.cccb.2026.100547)
Supplement: Supplementary file 1 [file mmc1.pdf]

SUPPLEMENTARY MATERIALS

DETERMINANTS OF CEREBRAL MICROBLEED PRESENCE AND BURDEN IN CADASIL

Authors :

Jessica Lebenberg[1,2], Louis Lambert[1,2], Laura Tintoré-Carbonell[1,2], Mohamed Saichi[1,2], Antoine Guillonet[3], Hugues Chabriat[1,2,4]

Affiliations :

1- Inserm 1127, Institut du Cerveau, ICM, F-75013, Paris, France
2-Centre Neurovasculaire Translationnel – Centre de référence CERVCO, DMU Neurosciences, FHU NeuroVasc 2030, Hôpital Lariboisiere, APHP, Paris, France
3-Service de Neuroradiologie, Hôpital Lariboisiere, APHP, Paris, France
4-Université Paris-Cité, France
Corresponding author : hugues.chabriat@aphp.fr

SUPPLEMENTARY TABLES

Supp Table 1: Variables associated with CMB emergence and burden in CADASIL across the 3 different models

	OR/IRR	2.5%	97.5%	p_value
MODEL 1 (DEMOGRAPHIC, BIOLOGICAL CO-VARIATES)				
**Absence / Presence**				
**Age**	68.25	18.40	253.17	**2.71e-10**
**HTA History**	2.62	1.58	4.35	**1.84e-4**
**SWI**	2.60	1.62	4.17	**7.12e-5**
Male Sex	1.69	1.06	2.67	2.60e-2
EGFr 1-6	0.68	0.43	1.09	1.06e-1
Count				
**Age**	10.33	2.08	51.20	**4.27e-3**
**SWI**	2.09	1.09	3.97	**2.55e-2**
Hypercholesterolemia Visit	1.89	0.99	3.62	5.30e-2
Hypercholesterolemia History	1.54	0.86	2.74	1.44e-1
EGFr 1-6	0.57	0.31	1.05	7.09e-2
MODEL 2 (DEMOGRAPHIC, BIOLOGICAL AND MRI CO-VARIATES)				
**Absence / Presence**				
**N Lacunes**	146.89	26.38	817.76	**1.23e-08**
**Age**	33.76	8.38	136.01	**7.47e-07**
**HTA History**	3.14	1.79	5.53	**6.69e-05**
**SWI**	2.58	1.56	4.29	**2.29e-04**
Brain Injury	2.69	0.98	7.38	4.40e-02
Hypercholesterolemia History	0.64	0.38	1.09	**1.05e-01**
EGFr 1-6	0.67	0.41	1.11	1.24e-01
N PackCigarettes Year	0.20	0.04	1.09	6.31e-02
Count				
**nWMH**	17.08	4.37	66.70	**4.40e-5**
**SWI**	2.40	1.34	4.29	**3.20e-3**
N Lacunes	2.17	0.79	5.94	1.33e-1
**Anti Platelets**	1.88	1.03	3.45	**4.07e-2**
Hypercholesterolemia Visit	1.59	0.92	2.76	9.42e-2
**EGFr 1-6**	0.43	0.25	0.73	**1.71e-3**
**BPF**	0.08	0.02	0.38	**1.19e-3**
MODEL 3 (DEMOGRAPHIC, BIOLOGICAL, MRI AND CLINICAL/COGNITIVE CO-VARIATES (BINARIZED VALUES)				
Absence/Presence				
**Age**	20.22	4.67	87.59	**5.80e-05**
**SWI**	2.55	1.53	4.25	**3.44e-04**
**Stroke**	2.53	1.50	4.26	**5.04e-04**
**HTA History**	2.44	1.41	4.24	**1.49e-03**
mRS ≥ 3	2.18	0.91	5.21	8.03e-02
**Gait Disturbance**	2.01	1.07	3.78	**3.08e-2**
**EGFr 1-6**	0.56	0.33	0.94	**2.97e-02**
Count				
**N Years Education**	34.37	9.73	121.41	**3.93e-08**
**N Lacunes**	4.57	1.85	11.30	**9.80e-04**
**MMSE ≤ 24**	3.18	2.00	5.04	**9.17e-07**
**Hypercholesterolemia Visit**	2.81	1.70	4.64	**5.53e-5**
**EGFr 1-6**	0.43	0.27	0.69	**4.65e-04**
**Migraine with aura**	0.30	0.14	0.67	**3.00e-03**

Supp Table 1 : Odd ratios OR (resp. incidence rate ratios IRR) obtained for the logistic – absence/presence – (resp. quasi-poisson – count –) regressions for the different models. Variables in bold font had significant association on the emergence or increase of microbleeds (p<0.05). Abb : SWI : SWI MRI sequence used to identified microbleeds ; N Years Education : number of years of education ; EGFr 1-6 : genetic mutation located in EGFr 1-6 ; N PackCigarettes Year : number of pack of cigarettes-years ; HTA History : history of hypertensive status of the patient (and/or hypertensor medication) ; Hypercholesterolemia History : history of hypercholesterolemia (prior diagnosis or statins medication) ; Hypercholesterolemia Visit : hypercholesterolemia at each visit (total cholesterol > 240mg/dL and/or low cholesterol > 160 mg/dL) ; Brain Injury : history of brain injury of the patient ; Anti Platelets : anti-platelets medication ; N Lacunes : number of lacunes ; nWMH : white matter hyperintensity volume normalized by the total intra-cranial cavity ; BPF : brain parenchymal fraction ; Stroke / Migraine with aura / Gait Disturbance :history of events ; mrS >= 3 : modified Rankin Scale binarized – threshold at 3 – ; MMSE <= 24 : Mini-Mental State Examination binarized – threshold at 24 –.

SUPPLEMENTARY FIGURES

Supp Figure 1: Acceptable clinical error

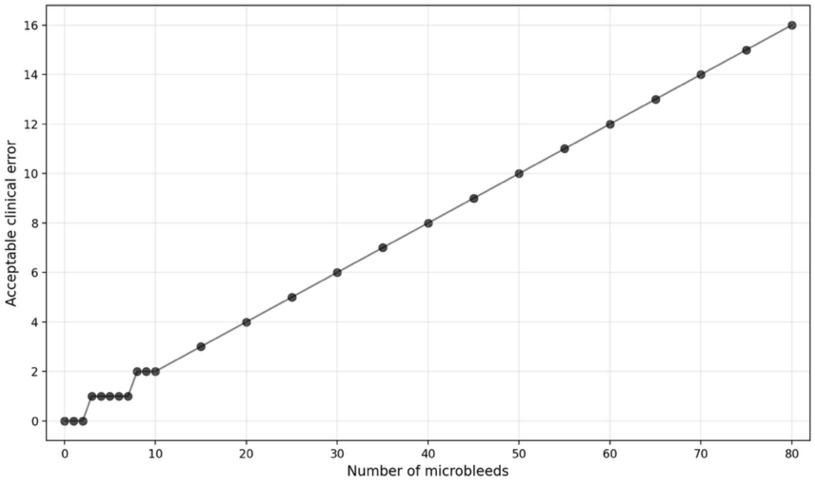

*Supp Figure 1**:** The acceptable clinical error was defined as 20% of the total number of identified microbleeds. For each count (N), the error was calculated as 0.20 × N, rounded to the nearest integer; this value defines the margins of agreement (i.e., if the calculated error = 4, the acceptable range is N ± 4). For example: 5 → 4–6; 10 → 8–12; 25 → 20–30.*

Supp Figure 2: Continuous CMB count comparison

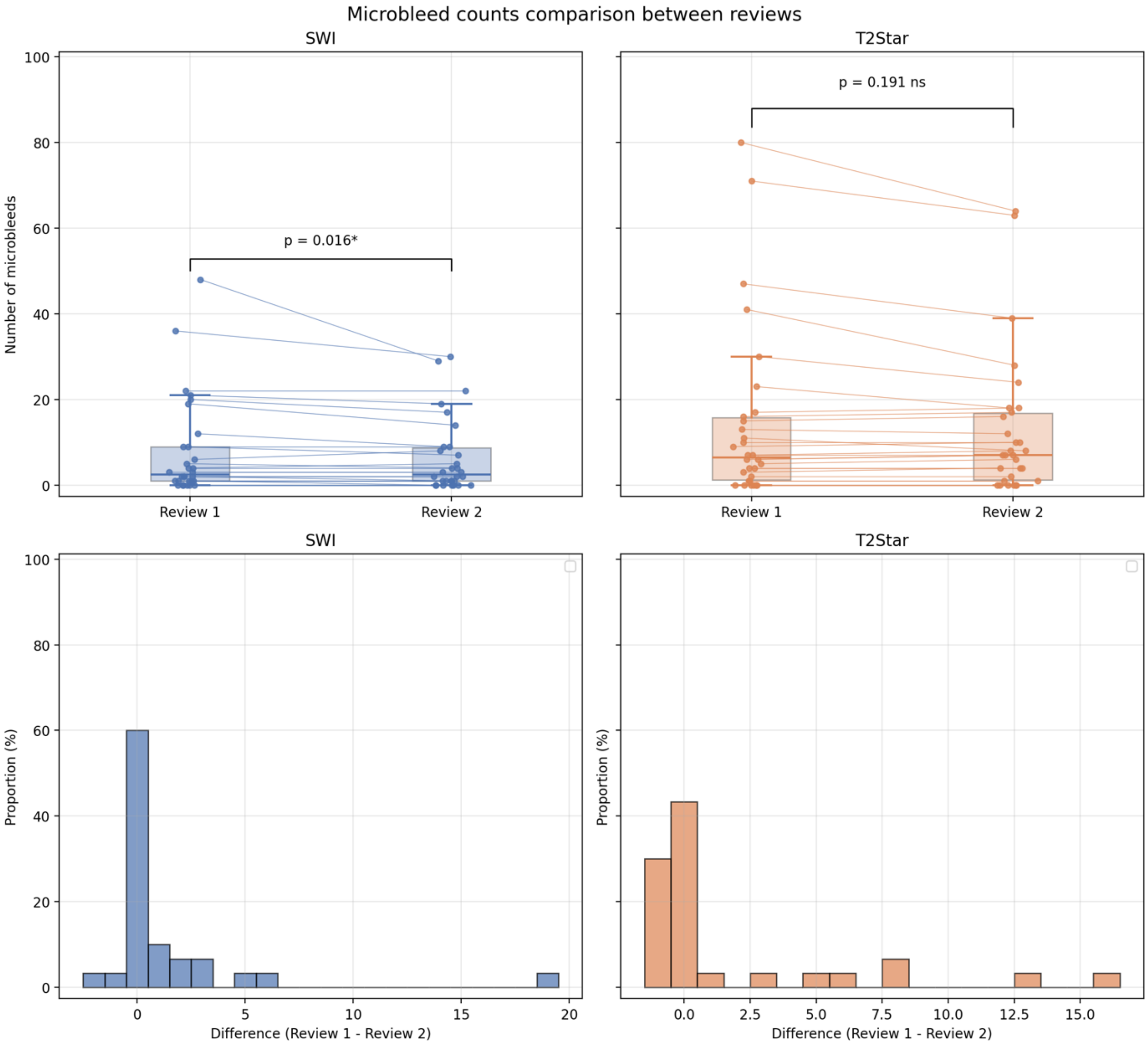

Supp Figure 2 : Paired counts of microbleeds in the two reviews of the reliability analysis (top line) for the SWI (left) and the T2star (right) sub-dataset. A paired Wilcoxon test showed that the difference of counting was non-significant for the T2star sub-dataset and sgnificant for the SWI sub-dataset (pvalue < 0.05). The bottom line displays the proportion of difference values. For the SWI sub-dataset, 60% of the calculated differences were null.

Supp Figure 3: Variables associated with CMB emergence and burden in CADASIL across the 3 different models

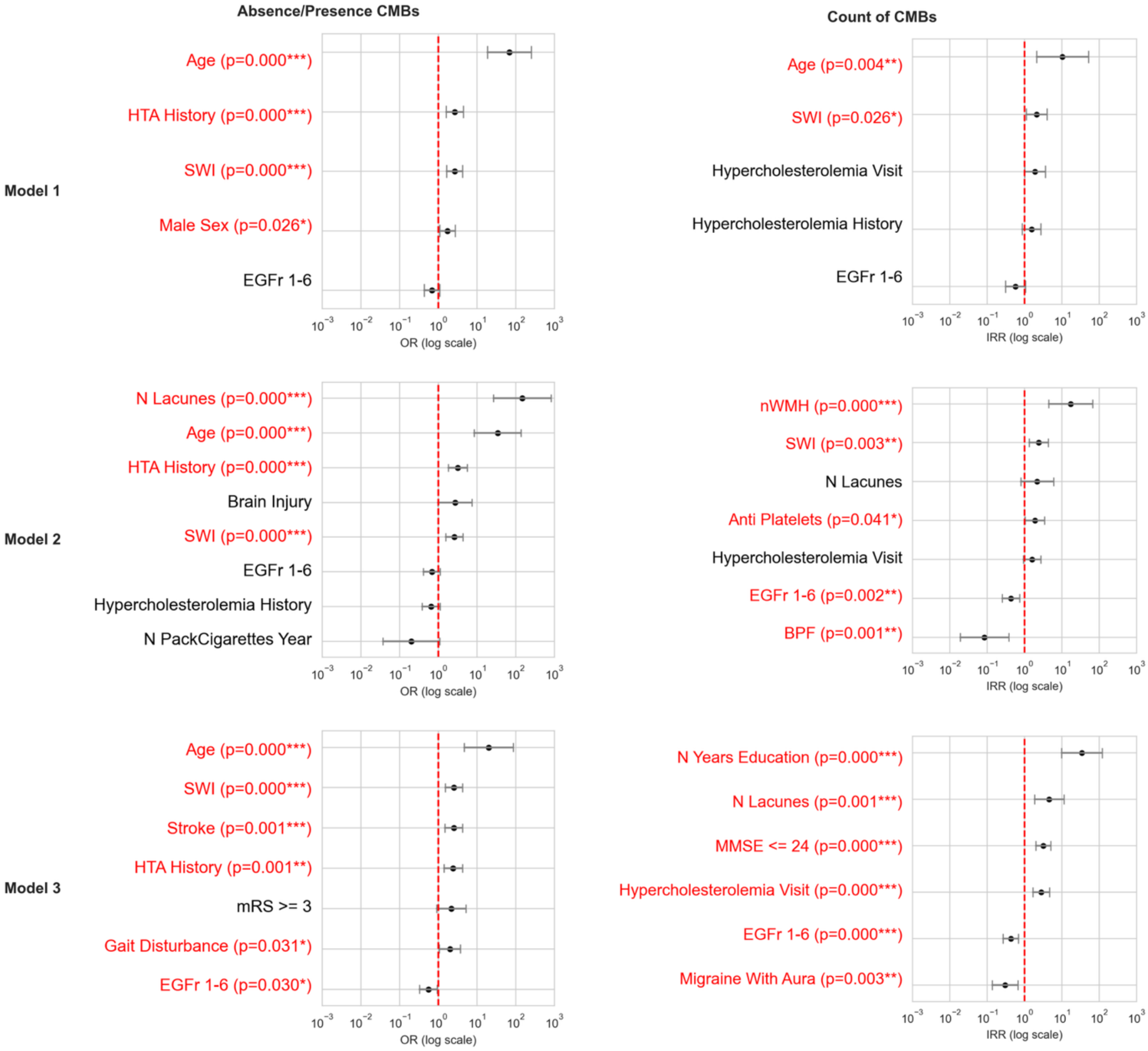

Supp Figure 3 : Forest plots of the odd ratios OR (left) and incidence rate ratios IRR (right) scores obtained for the logistic (absence/presence) and quasi-poisson (count) regressions in the frame of the different models. For a better visualization, results were plotted with a log scale. Variables in red had significant effect on the emergence or increase of microbleeds (p<0.05). Abb : SWI : SWI MRI sequence used to identified microbleeds ; N Years Education : number of years of education ; EGFr 1-6 : genetic mutation located in EGFr 1-6 ; N PackCigarettes Year : number of pack of cigarettes-years ; HTA History : history of hypertensive status of the patient (and/or hypertensor medication) ; Hypercholesterolemia History : history of hypercholesterolemia (prior diagnosis or statins medication) ; Hypercholesterolemia Visit : hypercholesterolemia at each visit (total cholesterol > 240mg/dL and/or low cholesterol > 160 mg/dL) ; Brain Injury : history of brain injury of the patient ; Anti Platelets : anti-platelets medication ; N Lacunes : number of lacunes ; nWMH : white matter hyperintensity volume normalized by the total intra-cranial cavity ; BPF : brain parenchymal fraction ; Stroke / Migraine with aura / Gait Disturbance :history of events ; mrS >= 3 : modified Rankin Scale binarized – threshold at 3 – ; MMSE <= 24 : Mini-Mental State Examination binarized – threshold at 24 –.

APPENDICES

Appendix A : WMH segmentation

As recommended in (Lebenberg et al., 2023), LST-LPA pipeline was used to segment WMH from native FLAIR acquired on the 1.5T GE scanner (resolution of 0.5x0.5x5.5 mm3) (see article Table 1) (Schmidt, 2017). Probability maps were threshold at 0.

BIANCA pipeline was used to segment WMH from FLAIR acquired on the 3T SIEMENS scanner (resolution of 0.5x0.5x1.0 or 1x1x1 mm3) (see article Table 1) (Griffanti et al., 2016). As recommended in (Lebenberg et al., 2023; Ling et al., 2018) FLAIR and T1w-MRI were considered as input for this pipeline. The T1w-MRI was linearly registered to the FLAIR MRI using a dedicated pipeline (Avants et al., 2009, 2011), and the signal bias was corrected for the both images (Tustison et al., 2010). To guaranty the homogeneous field of view between the training set and the data to be segmented required by the BIANCA guidelines, a FLAIR synthetic image (FLAIR template) was created using 20 FLAIR images from the CERVCO cohort. Both T1w- and FLAIR MRIs previously preprocessed were then linearly registered toward the FLAIR template. The intensities of FLAIR and T1w-MRI were scaled to match with patient data used to create the FLAIR template (the T1w-MRI was rescaled according to T1w-MRI associated to FLAIR involved in the template). Only voxels included in the brain tissue mask described in the article paragraph 2.1.3.1, were considered by the BIANCA pipeline. The data included in the training set described hereafter were similarly processed. The training set was composed by the 20 different CADASIL data including in the CERVCO cohort and with various WMH load (Lebenberg et al., 2023; Ling et al., 2018). Each output probabilistic map was thresholded using a data-specific value: a two-class K-Means clustering was applied to non-zero voxel intensities to identify the high-intensity cluster corresponding to WMH, from which descriptive statistics were extracted. The final threshold was then set to a fixed value of 1 when the mean cluster intensity was below 0.75, or otherwise to the mean intensity itself.

All masks were visually inspected and corrected, if necessary, by a scientific expert.

References

Avants, B., Tustison, N., Song, G., 2009. Advanced normalization tools (ANTS). Insight J.

Avants, B.B., Tustison, N.J., Wu, J., Cook, P.A., Gee, J.C., 2011. An Open Source Multivariate Framework for n-Tissue Segmentation with Evaluation on Public Data. Neuroinformatics 9, 381–400. https://doi.org/10.1007/s12021-011-9109-y

Griffanti, L., Zamboni, G., Khan, A., Li, L., Bonifacio, G., Sundaresan, V., Schulz, U.G., Kuker, W., Battaglini, M., Rothwell, P.M., Jenkinson, M., 2016. BIANCA (Brain Intensity AbNormality Classification Algorithm): A new tool for automated segmentation of white matter hyperintensities. Neuroimage 141, 191–205. https://doi.org/10.1016/j.neuroimage.2016.07.018

Lebenberg, J., Zhang, R., Chabriat, H., 2023. How to measure white matter hyperintensities in a long-term CADASIL cohort study? Presented at the Annual Meeting of the Organization for Human Brain Mapping (OHBM), Montreal, Canada.

Ling, Y., Jouvent, E., Cousyn, L., Chabriat, H., De Guio, F., 2018. Validation and Optimization of BIANCA for the Segmentation of Extensive White Matter Hyperintensities. Neuroinformatics. https://doi.org/10.1007/s12021-018-9372-2

Schmidt, P., 2017. Bayesian inference for structured additive regression models for large-scale problems with applications to medical imaging. (Thesis). https://doi.org/10.5282/edoc.20373

Tustison, N.J., Avants, B.B., Cook, P.A., Zheng, Y., Egan, A., Yushkevich, P.A., Gee, J.C., 2010. N4ITK: Improved N3 Bias Correction. IEEE Trans. Med. Imaging 29, 1310–1320. https://doi.org/10.1109/TMI.2010.2046908
